# Massive and Microscopic: Autoethnographic Affects in the Time of COVID

**DOI:** 10.1177/1077800420965570

**Published:** 2021-09

**Authors:** Anne Harris, Stacy Holman Jones

**Affiliations:** 1 RMIT University, Melbourne, Victoria, Australia; 2 Monash University, Melbourne, Victoria, Australia

**Keywords:** Critical autoethnography, affect, affect theory, meditation, mental health, COVID

## Abstract

This essay uses several of the prompts from the *Massive::Microscopic* experiment as a jumping off point for considering how affect theory and critical autoethnography offer us a framework for understanding, creating, and acting together in the time of COVID. Through stories of cloud-watching, mindfulness meditation, and other encounters with atmospheres and movements, we connect individual experiences of the pandemic to Buddhist understandings of a universal “we.” As a research practice committed to joining microscopic with macro lived experience, critical autoethnography offers a speculative method for collective reckoning with our infinitesimal selves in relation to the infinite of a pandemic.

The force of the affective moves us. When this movement tunes toward an experience that can be defined as such, the conscious share of the nonconscious has briefly made itself felt.

—Manning (2016, p. 20)

## Intimate Objects

Take photos of the 3 most intimate/familiar objects in your lockdown. What have you spent the most time with? Write a couple of paragraphs each from the perspective of each object.^1^

### Clouds

Every morning, before you check the news that’s washed into your feed overnight, you check the sky.^2^ You check the level of daylight streaming in through the window. You check the pane for signs of rain or frost. You check the clouds. It is winter in the southern hemisphere and in the months of June, July, and August, the Melbourne sky is mostly gray, a thick blanket of clouds punctuated by blue-sky-brilliant-white-cloud days that promise something other—something else. What? Another kind of day, one in which you find yourself outside and walking toward the horizon line they illuminate. Another time, one of becoming something else in light of what’s happening now. A sign and a time of hope.

Last night, you fell asleep reading Ali Smith’s *Spring*—the third in a series of seasonal works begun in the wake of Brexit. *Spring* tells a story in a “time of walls and lockdown” (A. Smith, 2019). It’s a story of how the time we’re living in—and through—is changing everything, including nature. And most certainly clouds.

In Smith’s book, the changing story of clouds (as well as how clouds change the story) is told through the work of visual artist, Tacita Dean. In the 1990s, Dean set out in a hot air balloon, intent on realizing a childhood dream to catch a cloud. But because hot air balloons can only ascend into the sky on cloudless days, she learned that it was “impossible to catch, keep or own a cloud” (A. Smith, 2019, p. 219). She became a cloud watcher instead.

When Dean moved to Los Angeles in 2014, the most surprising thing about her new home was the clouds. Driving down Sunset Boulevard early on in her stay, she “was confronted by a voluminous atomic cloud blooming at the end of the road in front of [her], back-dropped by a deep blue sky” (qtd. in Marian Goodman Gallery, 2016). She was inspired to make her cloud-watching into paintings made with blue chalkboard paint over photos of clouds, drawings made with white charcoal pencil, and works made with chalk and spit on blackboards.^3^ The collection of clouds she made in Los Angeles, exhibited in “A Concordance of Fifty American Clouds,” takes its title from *A Complete Concordance to Shakespeare*. Dean looked up the word cloud and “was delighted to find how variously and how richly the word appeared in his plays” (Marian Goodman Gallery, 2016).

**Figure 1.**
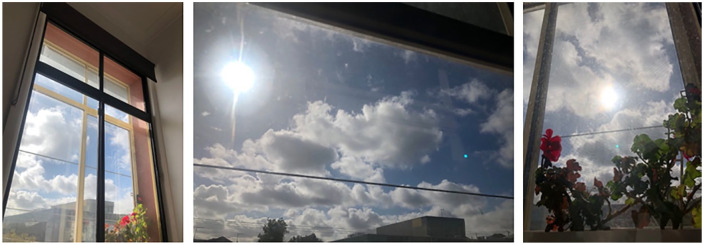
Clouds telling the story of now.

Shakespeare’s clouds stood in for a number of feelings and contexts that resonate with now: “anxiety, political instability, loss and uncertainty” (Engberg, 2016). And of course, for the sailors in Shakespeare’s plays—and everywhere—clouds are guides to navigating the weather and signs of “dangers [to] come” (Julius Cesar).

In this sense, clouds are *haecceities*, in Gilles Deleuze and Felix Guattari’s terms—harbingers of “thisness” (from the Latin *haec*, or “this”). Clouds-as-haecceities embody a “change between states, the becoming individual becoming different from what was before in a process of change or division” (Hougaard, 2012, p. 42). Deleuze and Guattari’s (1984) haecceities are modes of “individuation very different from that of a person, subject, thing, or substance” (p. 262). As modes of becoming, haecceities are the jumping off point for their consideration of affect. They write affect in terms of changes between states, such as the changing of a

season, a winter, a summer, an hour, a date have a perfect individuality lacking nothing, even though this individuality is different from that of a thing or a subject. They are haecceities in the sense that they consist entirely of relations of movement and rest between molecules or particles, capacities to affect and be affected. (Deleuze & Guattari, 1984, p. 262)

As an example of the affective force and capacities of relations of motion and rest between molecules, Deleuze and Guattari invoke the movement of time according to the microscopic and massive shifts of the personal. In other words, time moves in the affective gathering of molecules and matter like the weather—in the clouds, winds, and rainbows—of our worlds. Leaning on the fiction of Michel Tournier (1967), they write,

We can conceive of an abstract time that is equal for haecceities and for subjects or things. Between the extreme slownesses and vertiginous speeds of geology and astronomy, Michel Tournier places meteorology, where meteors live at our pace: “A cloud forms in the sky like an image in my brain, the wind blows like I breathe, a rainbow spans the horizon for as long as my heart needs to reconcile itself to life, the summer passes like vacation drifts by.” (Deleuze & Guattari, 1984, p. 262)

Living at your pace, and the modes of becoming now syncopating your days like the weather, makes the call of autoethnography feel apt as you look for ways to reconcile yourself to (this fractured, individualized, and atomizing) life. And perhaps this now-ness, this thisness, isn’t just for autoethnographers because we are all—each of us—reckoning with our infinitesimal selves, and relation to the infinite of a pandemic. That reckoning needs a form, a way of making sense and making something of selves in relation to worlds, and in relation to the others we miss—those who we long to touch alongside those whose absence is always political—those who are not “seen, acknowledged, and counted as present” (Barad, 2017, p. 113). These reckonings and longings are political questions, and they are questions for critical autoethnography (Harris & Holman Jones, 2019).

As we—Anne and Stacy—have written elsewhere, critical autoethnography is research and creative practice motivated by an abiding commitment to bring together the granular detail of the personal and the speculative power of theory in an effort to understand *how* “stories animate and become the change we seek in the world” (Holman Jones & Harris, 2018, pp. 1–2). Critical autoethnography is a mode of thinking, feeling, writing, and making that explicitly links the concrete and the specific (the thisness of our personal becoming) with critical theory’s commitments to social justice and making the invisible visible. These modes of making and commitments to action distinguish critical autoethnography as relational, rather than individual. As we-search, rather than me-search. It’s how you “assemble a we”: a community of thinkers, makers, and doers joined in their commitment to “speaking and embodying a collective” message, or “will,” after Judith Butler (Butler, 2015, p. 156; Holman Jones, 2017).

Critical autoethnography is a field for the exchange of the massive and the micro, presences and absences, negative and positive charges. It’s like the charge between earth and a storm cloud. It’s like Dean’s “Storm Clouds,”^4^ which she made as a harbinger of the political storm of Brexit. Dean later said she made the work “far too gentle. I think it should be way, way more angry” (Brown, 2019). That’s the thing about storm clouds—we ask them to hold so much: our anger, fear, and anxiety, and also our desire, light, and electricity.

Karen Barad (2015) writes about storm clouds in her essay on transmaterialities in/as lightning.^5^ In a sequence of charged moves, the cloud and the ground send out tiny electrical signals—what Barad calls “material imaginings”—of a “desiring field that animates [the] intra-active becoming” of earth and sky (p. 409). So, while we like to think of lightning issuing from the heavens in the gathering pressure of a storm, the striking thing about lightning—and autoethnography—is how it connects cloud and ground (self and world) in an atmosphere of intimate indeterminacy. And that’s one thing we’ve got a lot of right now—the striking connections of self and world in an atmosphere of indeterminacy.

You move in the extreme slownesses and vertiginous speeds of now, your capacity to affect and be affected taken down to the microcosm of home—you, your chairs, your dogs, your mindfulness meditation podcasts, your sea. Your window, through which you watch the sun rise and set, marking the changing seasons in the shape and color of the clouds. And though you wish you could ride in a hot air balloon looking for signs of better weather, you stay put. You stay inside watching the clouds and wondering about the capacity of matter, air, and energy to affect and be affected.

### Christmas Trees

COVID is not the only reason you’ve had cause to isolate. This thing is unprecedented, sure, but its enactments are not. A breakup, a lost job, moving to a new city. A slipped disk, writing a new play/book/film, despair. Many moments in life when you’ve chosen to be alone, to retreat, to repair in seclusion. You remember these past isolations as COVID unfolds. You remember that staying put is not stasis, physically nor emotionally. It is also not cause and effect—virus causes isolation, or isolation causes illness. Volitional movement, which is what’s required if your actions-as-causes are to have the effects you intend, as Erin Manning (2016) writes, is a “play-by-play” fiction you write “after the fact” (p. 19). This “post facto narration” is “usually how we explain our actions, but it is not how we act” (Manning, 2016, p. 19). Manning’s writing on affect and attunement suggests another view on how we act, one

based on a continuous interplay on conscious and nonconscious movement with nonconscious movement playing a vital part, especially as regards movement’s creative potential. (Manning, 2016, p. 18)

Movement’s creative potential brings you back to your body as it habitually moves toward, along and away from the beach on the meditation walks you take every morning and afternoon. Meditation’s movement reminds you how time folds and the folds touch each other, memories falling into the dip between the folds. July is winter here, and after 23 years of living in the southern hemisphere, you still catch yourself thinking it’s November or December, because those are “cold” months in North America where you are from. Yesterday, someone said, “It feels like Christmas.” And it does. So, you begin attending to sensory signals of Christmas-in-July during the “directed movement” of your daily mindfulness walks and you start to notice trees with Christmas baubles all around and above you.

What does it mean to feel Christmas in the plane trees that dominate your lockdown sky? The trees are, like so many other elements in your bayside environment, grand and beautiful; solid and old. They are also non-native plants that cause intense allergic reactions, and for these reasons, they are being replaced by your local council with native Moreton Bay figs, jacarandas, and gum trees. None of these kinds of trees populated your childhood New York landscape. But there is a feeling you have with them, a connection folds experience back on itself so they feel like old friends who are beloved even as they irritate and make your eyes weep. Your meditation walks and your connection with the plane trees are what Manning (2016) describes as *déjà-felt*:

In our everyday movements, especially in relation to movements that have become habitual, a movement might nonetheless feel completely volitional. When this is the case, what has happened is that we’ve experienced a sense of déjà-felt, in the event. This déjà-felt occurs in the interstices of the conscious and the nonconscious, directing the event to its familiarity-in-feeling. (p. 19)

The déjà-felt under COVID isolation makes your walks *feel* completely volitional. The plane trees are your familiarity-in-feeling during this time, your non-virtual but not-necessarily-corporeal touchstones. Touch-trees. Christmas-baubles-in-July trees. You-are-still-here-trees.

**Figure 2.**
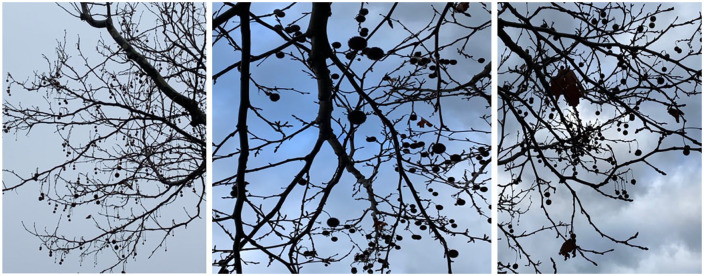
Déjà-felt Christmas trees.

## Ice Cube as Universe

Observing, sensing, sensemaking: You’ll need an ice cube.

Get an ice cube. If comfortable for you to hold it in your hand, do this, and keep it there until it melts (if not comfortable, it’s fine to place it somewhere it will melt). Don’t look away. When it’s gone, write for 15 minutes without stopping your typing on a keyboard or lifting the pen from the page. You have 24 hours to complete this task.^6^

**Figure 3.**
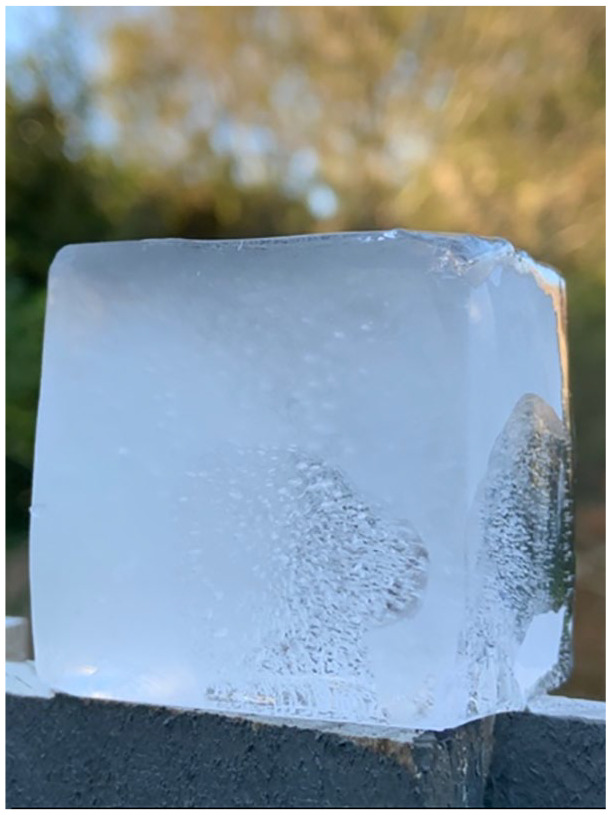
Ice to water, water to gas.

### Ice to Water

The self-soothing skills of Marsha Linehan’s (2015) Dialectical Behavioral Therapy (DBT) training help you with the stresses of COVID. You joke with a friend who also suffers from emotional dysregulation that for once, your disorder is an advantage in coping with daily life, rather than a disadvantage. You walk and walk and walk and your meditation becomes *ice to water; water to gas.* For Ben Anderson (2014) (extending Deleuze), affective atmospheres can be traced through the strange materiality of “the element of air or the state of a gas [which] reminds us that atmospheres are material phenomena,” expressing something “remarkable for how they affirm the singularity of this or that atmosphere and the inseparability of the atmosphere from that which it emanates from” (p. 140).

DBT is a powerful set of skills which assist sufferers of emotional dysregulation by teaching us distress tolerance and radical acceptance. It is a combination of acceptance skills and change skills (hence, the “dialectical” nature of the therapy). The core activities use the acronym TIPP, which stands for: (T) temperature change: cold shower, dunking your head into cold water, holding an ice cube; (I) intense exercise like sprinting, jumping jacks, or other activity guaranteed to raise your heart rate; (P) paced breathing to slow your heart rate; and (P) paired muscle relaxation, such as clenching and releasing. Emotional dysregulation is a disease of feeling cut off, and temperature change and the ice cube become totemic images for this pandemic, and your disorder.

It is the ice cube that holds your attention. It is the ice cube that also appears in Buddhist meditation:

Right here, right now.

Be absolutely still and notice . . . the experience of pure awareness . . . without resistance.

This attention is the universal attention. It has no boundaries . . .

Soon you will see it is your home.

It is your essence.

Like an ice cube on the sea, you melt into this ocean

and there’s only bliss . . . this bliss of freedom . . . of homecoming. (Brach, 2019)

**Figure 4.**
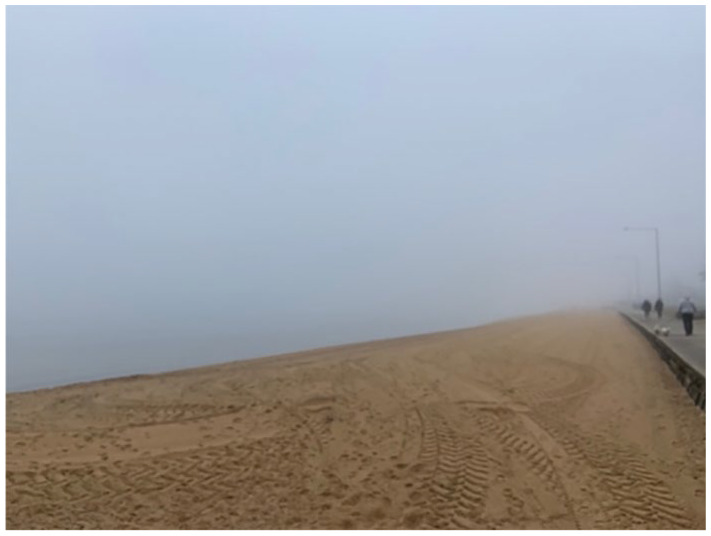
Remember the ocean.

You walk, listening to Buddhist mindfulness teacher and psychologist Tara Brach. The rhythm of your footsteps, the water, Tara’s voice, the weather, your growing attention to sensory presence and following your breath, are haecceities—movements of becoming that help your days go smoothly. You like the ice-cold rain on your face, getting out of your head, the walk drawing you into your body through shifting temperature, pace, and sensory attunements. When Brach talks quietly, air-podding into your ears about the ice cube melting, it’s more than a metaphor for separateness and togetherness. She says,

Just as a bright sun causes ice cubes to melt, in the moments when we feel connected and kind, we create a warm environment that encourages others around us to relax and open up. Each time we widen the circle of caring—with a smile, a hug, a listening presence, a prayer—the ripples flow out endlessly. (Brach, 2003, p. 242).

She tells you, “There is no them; there is only us” (Brach, 2018). She tells you, “If you can remember you are the ocean, you can happily weather the waves; if you forget you are the ocean, you’ll be seasick every day” (Brach, 2018).

Brach and mindfulness work draw your attention out to sea: you walk and gaze at the water, which changes mood like you do. You are the ocean, not the waves. You are the whole beach, the bay, the ocean, nature. You belong. Belonging is bigger than any small story you have of yourself. You is a story you tell yourself. And now, another story is emerging. This is how critical autoethnography is also a mindfulness practice: connecting the individual self—the I and the you—with the we. A we is timeless and limitless, a material imagining that is at once massive and microscopic. Melt the ice cube and become one.

### Sleeping Not Swimming^7^

There are a number of people swimming in the bay today without wetsuits. You’re amazed at how many. You can’t even get into your kayak in a wetsuit in winter, you’re so averse to the cold. Still, you walk along the beach and imagine yourself a much tougher, and wiser senior swimmer. You think the cold won’t bother you so much when you’re older. You’ll get up early every morning, feeling excited about starting the day with a dip in the ocean. You imagine yourself a person who does it because you want to, and it’s enjoyable. You become someone who would rather swim than sleep.

You strive to stay present on your walks to the beach, either attending to the sensory landscape or mindfulness meditations. Or both. A packet at the water’s edge shines in the sunlight, pulling your attention. Aspirin?

**Figure 5.**
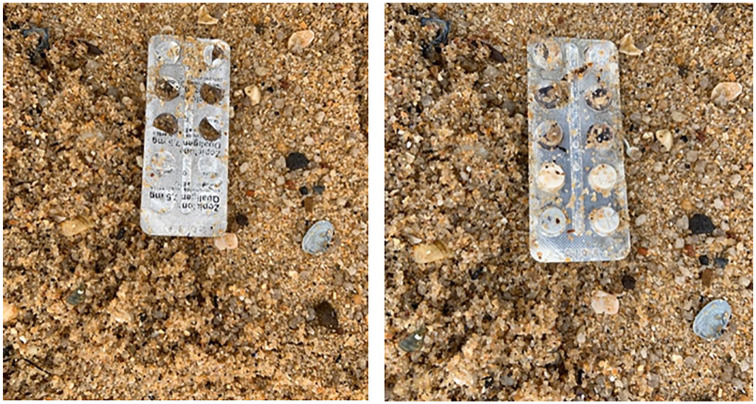
Sleeping, not swimming.

**Figure 6.**
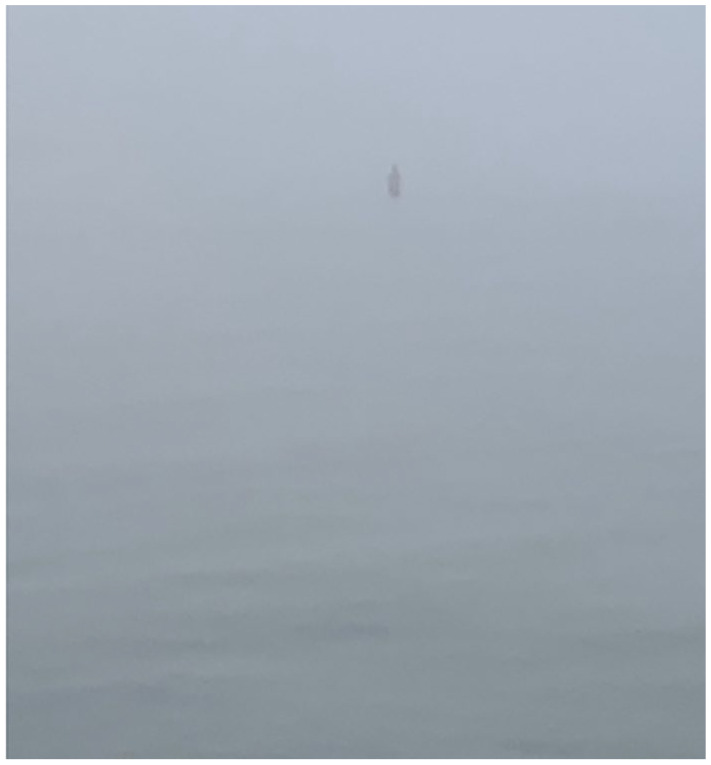
Winter swimmer, not sleeping.

You break your rule and take a photo, front and back. When you get home, you look it up: *Zopiclona Qualigen*, a powerful sleeping pill. COVID is causing anxiety, feelings of fear, and powerlessness. Sleeplessness. You’re glad for your mindfulness meditation along the ocean’s edge. You’re glad for this writing and how each practice helps you feel and understand that life is always changing and how thoughts pass like waves. Brach teaches you about the waves of change called *anicca*, and that in Buddhism,

changeability is one of the perennial principles of nature. Everything changes in nature and nothing remains static. This concept is expressed by the Pali term *anicca.* Everything formed is in a constant process of change (*sabbe sankhara anicca*) . . . In nature there are no static and stable “things”; there are only ever-changing, ever-moving processes. Rain is a good example to illustrate this point . . . [R]ain is nothing but the process of drops of water falling from the skies. Apart from this process, the activity of raining, there is no rain as such which could be expressed by a seemingly static nominal concept. The very elements of solidity (*pathavi*), liquidity (*apo*), heat (*tejo*) and mobility (*vayo*), recognized as the building material of nature, are all ever-changing phenomena. (de Silva, 2005, n.p.)

You imagine the person who was in possession of the *Zopiclona Qualigen* walking down to the beach because they can’t sleep, and sitting on the breakwater, buffeted by cold winter wind, watching the horizon, listening to the rhythm of the waves, slowly returning to their body. They stand up to go home as the sun sets clear and red on the winter water, walking up the boardwalk into that glow, and as they rise, the pills they have clutched tightly in their COVID hands slip unnoticed onto the sand. They go to sleep a short time later, calmed, not yet realizing that their drugs are gone and they haven’t noticed, swimming in the ocean of their exhausted body.

## Desperation Is Energy and Energy Can Move Things

[I am] A fool who believes that death is waste and love is sweet and that the earth turns and men change every day and that rivers run and that people wanna be better than they are and that flowers smell good and that I hurt terribly today, and that hurt is desperation and desperation is—energy and energy can move things . . . (Lorraine Hansberry, The Sign in Sidney Brusteins’ Window)

How has COVID made you or someone you know better than you were? How has the desperation of COVID 19 become a kind of energy for you that can move things . . .? (Hansberry, 1965)^8^

### Cueing Movement

Your back hurts, your neck’s sore, and your brow is permanently furrowed. In the screen reflection of Zoom, you look like you have tears staining your face, etched by the water of a thousand mountain springs over rock. You are calcifying; you are a statue, stone-still.

Erin Manning (2016), in *The Minor Gesture*, distills our relationship with sitting in learning, in ways not unfamiliar to teacher-researchers:

Most of our education systems are based on starting from stillness. We learn in chairs. We associate concentration with being quiet. We discourage the movement of thought we call daydreaming, particularly in the context of “learning.” We consider the immanent movements of doodling to be a distraction. We are told not to fidget . . . (p. 122)

You set out on a walk, noticing everything that is sensorial and atmospheric: the gum leaves are gray-green; the water is muddy; the sky is a white, thick cloudbank, the kind of sky-becoming-snow sky, though snow never comes here in Melbourne—crested cockatoos squawk; two black swans paddle silently by with graceful nonchalance. Starting from movement, mindfulness practice is a focus, rather than a distraction. It is an investment in the becoming of movement, in what Manning (2009) terms “readiness potential”: the “immanence of movement moving [or] how movement can be felt before it actualizes” (p. 6). Your pace is modulated by these movements, your body is attuned to what you see, smell, touch, hear, taste. Your body moves in relation to.

**Figure 7.**
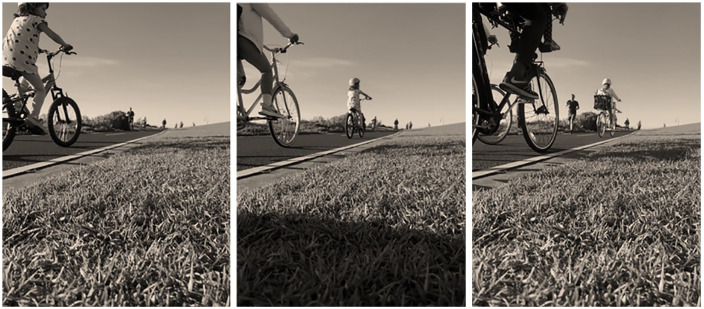
Collective movement happens in the moving.

Along the water, joggers weave in and out of walkers. Walkers with babies go slower, walkers with dogs go erratically. The water moves toward the shore and you orient your body to the lap of the waves. You like that the water has not been taken away from you (yet) by the isolation shutdown. You like the way people on the boardwalk and bike path which runs parallel are aware of each other and move while talking, listening, and looking; not consciously but in anticipation; you like how we gather and disperse kinaesthetically, a collective choreography. Erin Manning (2016) calls this collective movement *cueing*:

The choreography of collective movement is made possible by the interrelation between the intervals the movement creates and the collective capacity to cue and align to them, in the moving. Cueing is an important activity in the relational field. It is not directly tied to volition or intentionality. It happens in the moving. Although it may feel like it is individuals cueing to one another, what is actually happening is that movement is cueing to a relational ecology in the making. (pp. 120–121)

You like how this links to what you think is “actually happening” in critical autoethnography. You see that work as cueing movement that offers its participants a becoming that is a relational ecology, even and especially in times of pandemic isolation. Its attention to the micro (self) and the macro (culture) invites a choreography that makes possible activity in a relational field that remains even as the cueing changes, as the waves rise and fall away.

### Energy Can Move Things

For many of us, the time of COVID is a time of powerlessness and fear; storm clouds gathering on the horizon. It is a time of fatigue. Zoom fatigue. Scarcity fatigue. Presser fatigue.^9^ Still, this fatigue buzzes; it’s full of movement.

It’s hard not to see fatigue and movement as a painful binary. But you try, in an intentional, mindfulness kind of way, to see movement and fatigue as a continuum, like waves on the ocean and the turning of the seasons. No ending or beginning, but one folding into the other. And you try, too, to forget volition or intentionality and see movement and fatigue as intra-active, relational activity that happens in the moving. To see not them and they, only us and we.

Your sister, Achol, calls. It’s a cold Sunday evening in Melbourne, and the state government announces an unprecedented “hard lockdown” on public housing flats which are home mostly to refugee-background Australians, many from African countries. These are people who have already experienced enormous trauma before arriving, and for most of them, after arrival too. Achol’s call—the desperation in her voice and her words, which prove as false the information on the evening news about support and supplies—moves you into action. Like Hansberry says, desperation is active and urgent. It has energy.

You and others mobilize quickly to bring food and other supplies. Not a massive mobilization that will address the systemic racism and classism that allows for this kind of police action directed against the most vulnerable, but a micro-mobilization that brings nappies just before they run out. Collective experiences of fatigue, the kinds that COVID makes, can also *move us.* Things and economies and cultures move in unexpected and sudden ways, beyond ourselves and our isolation. Move us beyond into a gathering of affective intensities, emergence—or eruption—of energy. Even when we feel terrible. Even when—especially when—we are hurting.

The supplies are not the whole of it. Achol has been through a lot, more than most of us can even contemplate. She bears that suffering stoically. But this is different. Three of her children, at her aunt Akech’s for the day, are now not able to get home. All the hurt, rage, and confusion borne of civil war in South Sudan suddenly resurface. English-only explanations blare through squawky loudspeakers. Dozens of buses of riot-geared police officers circle the towers of low-income flats like the residents are a terrorist threat. Life feels like the camps again.

Post-traumatic stress disorder (PTSD) assumes a temporality that is false; there is no post, and trauma has no past. It is a haecceity—a time where meteors live at our pace. On the third day of lockdown, some activists tie up banners around the grounds that say, “We see you” and “We are with you,” but Achol and other residents hold up signs in their (sealed) windows that say, “Help” and “We are not criminals,” putting language to a need to be part of something, rather being looked at or after.

You remember that the difference between illness and wellness, as Tara Brach puts it, is “I” versus “we.”

I/llness

We/llness

And breaking lockdown, even and only virtually, is still breaking, because we are all in this together, alonetogether. Energyandhope.

Driving toward the high-rise flats, you see the flashing lights illuminate the night sky a mile off. You think of Francis Alÿs site-specific work, “When Faith Moves Mountains.” Conceived in the last months of the Fujimori dictatorship in Peru in 2000, when the streets of Lima were filled with clashing bodies and a resistance movement was forming, Alÿs’ work was a movement that embodied the epic response needed to meet the present. He recruited 500 volunteers to move a sand dune on the edges of Lima, shoveling in unison for an entire day. They moved the dune 4 inches (10 cm), and their labors, barely evident in the moment, were invisible the next day. Alÿs said it was “a desperate situation calling for an epic response” (Alÿs, 2002). The idea that inspired the action was, “maximum effort, minimal result” (Alÿs, 2002). Even where “minimal change was effected, and only by means of the most massive of collective efforts,” the collective movement moved a mountain (Alÿs, 2002). Even, if only, for a moment.

**Figure 8.**
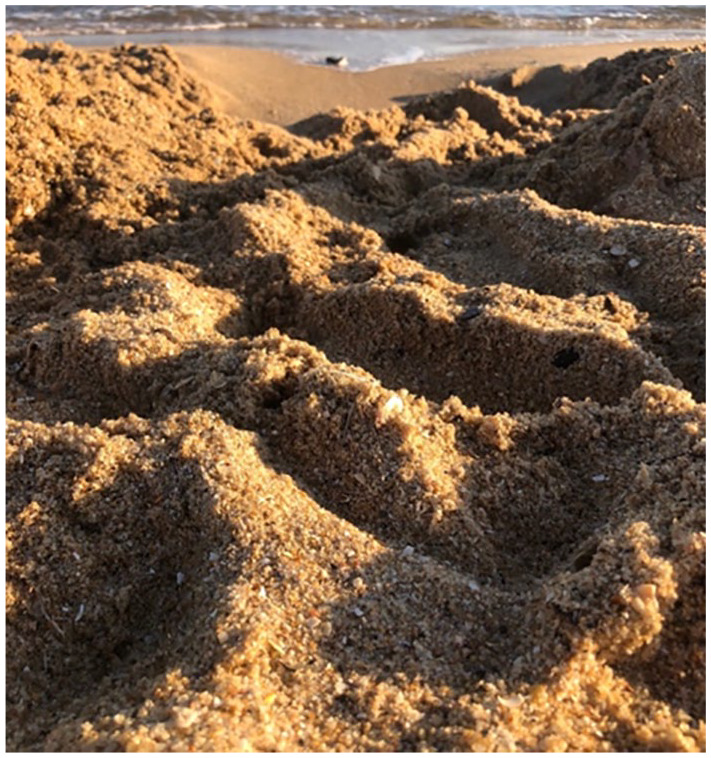
Maximal effort, minimal result.

We might be inclined to say, what’s the use of bringing bread and nappies and yes cigarettes and alcohol into the middle of a police-patrolled lockdown? What exactly is required to make change when you’re trying to move systems and people and practices that don’t want to change, that feel immovable? When you are infinitesimal selves, in relation to the infinite of a pandemic? As Sarah Ahmed (2019), in her book *What’s the Use?*, reminds us (as if we need reminding):

The more you are blocked the more you have to try to find a way through. The less support you have the more support you need. We might become each other’s resources, we prop each other up, because we understand how diminishing it can be to have to fight for an existence, to have to fight, even, to enter a room. Perhaps the harder it is to be, the more use you have for use. (p. 223)

Our efforts to be each other’s resources, to prop each other up, don’t need to be maximal effort for minimal results. We act in whatever ways we can, “trying to change the impossible, to move things an inch at a time all those thousands of miles toward the possible” (A. Smith, 2019, p. 274). We act because we see how making our commitments to action, no matter how small, is central to our becoming, *now*.

These micro acts are what Manning calls minor gestures, nearly imperceptible movements that can transform our ways of relating. Like critical autoethnography, minor gestures are speculative and improvisational while being pragmatic and “singularly connected to the event at hand” (Manning, 2016, p. 2). They are affective, material imaginings that “invent new forms of existence, and with them, in them, we come to be” (Manning, 2016, p. 2). Autoethnographic affects in the time of COVID use the fear, anxiety, fatigue, and desperation we are experiencing as an energy that can move things. They are minor gestures inventing new ways to be better than we were, before, for each other.

## Notes

1. *Massive::Microscopic* Prompt #2: Photos of intimate objects.

2. We—Anne and Stacy—have written this essay using a combination of second person voice, which highlights autoethnography’s relational focus, by calling you—the reader—to both witness and also act with the stories presented here (Holman Jones et al., 2013, p. 25) and to indicate a general “‘you’ who is all of us” (Gutkind, 2008, p. 124). We also use a first-person plural “we” to refer to the community of critical autoethnographers—a community that includes us (Anne and Stacy), you, our reader, and the assembly of we, critical autoethnographers (Holman Jones, 2017). In the words of mindfulness practitioner Tara Brach, in critical autoethnography, “There is no them; there is only us” (Brach, 2018). We invoke this collective we as a call to action and to share a message of hope.

3. You can see Dean’s cloud works here: https://www.wallpaper.com/art/in-the-clouds-tacita-dean-looks-to-las-skies-for-her-latest-body-of-work

4. You can see the work, originally titled “Foreign Policy,” here: http://www.artnet.com/events/museums/tacita-dean-landscape-at-royal-academy-of-arts/

5. This essay, published in *GLQ: A Journal of Gay and Lesbian Studies*, is itself an example of critical autoethnography, in how Barad links her work in quantum field theory with a story of lightning.

6. *Massive::Microscopic* Prompt #7: Ice cube as universe.

7. See the 1957 poem by British poet Stevie Smith “Not waving but drowning” as well as the 1980s Melbourne-based band *Not Drowning, Waving.* Both reference human relationships with nature and the environment.

8. *Massive::Microscopic* Prompt #20: Desperation is energy and energy can move things.

9. In Australia, the “presser” is the daily press conference during which the previous day’s COVID infection and death tallies are presented. They occur at mid-day, every day, though there are “leaks” about the coming numbers all through the morning and analysis of what the numbers signal for the next day, and the next stage of restrictions, all through the afternoon.
